# Implementing whole genome and transcriptome sequencing for cancer patients in routine healthcare: a comprehensive guide to costing

**DOI:** 10.1038/s41416-026-03422-0

**Published:** 2026-04-07

**Authors:** Christian Altbürger, Michael Menzel, Susanne Beck, Katja Lorenz, Marie-Luise Brygider, Carolin Ploeger, Andy Kahles, Markus Ball, Martina Kirchner, Fabian Schnecko, Andreas Jung, Frederick Klauschen, Tobias Grob, David Horst, Daniela Hirsch, Matthias Evert, Peter Schirmacher, Jan Budczies, Daniel Kazdal, Albrecht Stenzinger

**Affiliations:** 1 Institute of Pathology, Heidelberg University Hospital, Heidelberg, Germany; 2 Center for Personalized Medicine (ZPM), Heidelberg, Germany; 3 Institute of Pathology, Ludwig Maximilian University of Munich, Munich, Germany; 4 Institute of Pathology, Charité - Universitätsmedizin Berlin, Corporate Member of Freie Universität Berlin and Humboldt-Universität zu Berlin, Berlin, Germany; 5 Institute of Pathology, University of Regensburg, Regensburg, Germany; 6 Bavarian Center for Cancer Research (BZKF), Regensburg, Germany

**Keywords:** Cancer genomics, Tumour biomarkers

## Abstract

**Background:**

Molecular characterisation of solid tumours contributes to a precise diagnosis and can uncover a range of drug targets and biomarkers, improving patient management. Whole genome sequencing (WGS) combined with whole transcriptome sequencing (WTS) is the most comprehensive approach in molecular cancer diagnostics and is therefore becoming increasingly integrated in clinical care. A critical challenge in implementing WGS/WTS as a clinical-grade test is its high cost compared to targeted sequencing approaches, prohibiting wider use and access.

**Methods:**

To dissect this limitation, we performed a micro-costing analysis of an established WGS/WTS workflow based on short-read sequencing technology. We categorised the costs as follows: consumables, equipment and maintenance, personnel, and data processing/storage. We considered various scenarios, including sample volumes, personnel and equipment redundancy, inflation and mean coverage.

**Results:**

We have developed a multidimensional costing model that identified consumable costs, particularly flow cells, as the main cost drivers. While personnel and equipment costs decreased with larger case volumes processed (scaling effects), consumable and data processing/storage costs did not change significantly.

**Conclusions:**

Thus, falling prices of consumables would facilitate the broad implementation of WGS/WTS as a clinical-grade test.

## Background

In modern cancer medicine, which is becoming increasingly personalised, precise diagnosis is paramount for treatment guidance. A central aspect is the identification of predictive biomarkers and drug targets. Over the last two decades, the field of molecular pathology has advanced from tissue-based detection of variants in single genes to NGS-based detection of variants in either tissue or liquid biopsies utilising large gene panels [1, 2] or even whole exome (WES) [3] and whole genome sequencing (WGS) in dedicated clinical research programmes and networks [4]. This so-called comprehensive genomic profiling (CGP) enables the simultaneous analysis of most disease-associated gene variants that are targets for drugs, but also the characterisation of complex biomarkers, such as tumour mutational burden [5], microsatellite instability [6], and homologous recombination deficiency [7]. Compared to larger gene panels, WGS enables the most comprehensive detection of complex biomarkers and facilitates a more precise measurement of structural tumour genome variants [8]. Therefore, WGS has been widely used in cancer research, resulting in the publication of large genomic landscapes of various cancer types [9, 10] and providing novel insights into tumour biology, e.g., the identification of mutational signatures allowing the assignment of specific mutational patterns to their underlying molecular cause [11, 12]. In principle, these signatures can also be detected using WES, but WGS offers a higher resolution, particularly at low mutational burden [13]. In recent years, several countries have started integrating WGS into clinical care, primarily via specialised research programmes and networks. Based on the concept of a learning healthcare system, rigorous and systematic collection and integration of molecular data with clinical data not only facilitates the generation of real-world evidence leading to new clinical trial concepts, but also fuels basic research [14–24]. However, one of the main hurdles for the broad implementation of WGS in clinical routine care and reimbursement by healthcare payers is the high costs [8, 25]. These costs have been subjected to detailed analyses (micro-costing) for a variety of applications and healthcare systems during the last decade [26–31]. Rapid changes in and continuous development of sequencing technologies hinder the comparison between micro-costing analyses published so far and a comprehensive and detailed costing guide is missing. To fill this gap, we performed a micro-costing analysis of WGS in combination with whole transcriptome sequencing (WTS) designed for a clinical routine workflow. We rigorously analysed the cost parameters for tissue-based WGS/WTS, based on short-read sequencing technology, divided into the following categories: consumables; equipment and maintenance; personnel; research and development; computational data analysis/storage. Moreover, we computed various scenarios that contribute to costs, including the sample volume, different sequencing devices, genomic coverage/read depth, library protocols, redundancy for personnel and equipment, and inflationary/deflationary macroeconomic settings. We chose increased mean coverage as a scenario as it enables more accurate and sensitive variant calling and more reliable biomarker detection. To support individualised planning, we also developed a cost calculator that can be accessed online (https://hd-molpath.de/WGS-cost-calculator).

## Methods

### Micro-costing design

**Fig. 1:**
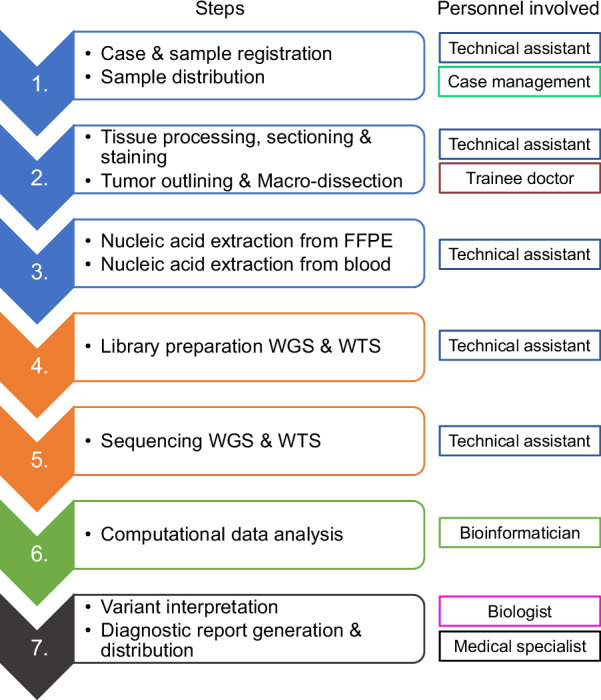
Diagnostic workflow for WGS/WTS. Schematic overview of the workflow for whole genome (WGS) and whole transcriptome (WTS) sequencing from sample reception to the generation of the diagnostic report and personnel involved in each step (case management, technical assistant, trainee doctor, bioinformatician, biologist, and medical specialist). The first three steps (in blue) comprise sample registration, tissue processing, and DNA and RNA extraction from the FFPE tumour tissue, as well as DNA extraction from blood as a patient reference sample. Steps four and five (in orange) comprise library preparation for WGS and WTS, followed by sequencing. Step six (in green) involves the bioinformatic processing of the raw sequencing data and computational analysis, such as variant calling. Step seven (in dark grey) involves a biologist interpreting the variants identified computationally and subsequently generating a diagnostic report.

For defining the diagnostic workflow for WGS/WTS, we determined the steps from sample reception to diagnostic report generation (Fig. 1). The wet lab workflow begins with tissue processing, which comprises formalin fixation and paraffin embedding (FFPE) of the tissue, slicing of the FFPE-tissue block and hematoxylin and eosin staining. These slides are used for annotating the region with the highest tumour purity (tumour cell content) by a trained pathologist and subsequent macro-dissection. Nucleic acids (DNA and RNA from the FFPE-material, DNA from blood) are extracted and used for manual WGS and WTS library preparation. We defined two library preparation protocols, one with mechanical fragmentation and one with tagmentation. These libraries are sequenced using short-read technology with an Illumina NovaSeq X Series 25B flow cell (300 cycles) (Illumina, Inc., San Diego, CA, USA) on an Illumina NovaSeq X Plus sequencing platform. The generated raw data is processed using a local Illumina DRAGEN setup (Fig. S1) and both raw and processed data (FASTQ, BAM and VCF files) are stored locally on storage servers. The computational analysis pipeline utilising local DRAGEN servers comprises the following steps: demultiplexing of the raw data, mapping of the FASTQ files, germline variant calling, and calling of somatic variants, copy-number alterations and complex biomarkers. Functional and clinical interpretation of the called variants are performed by trained and experienced molecular biologists, resulting in the generation of a diagnostic report, which is finally validated by a pathologist and distributed to the clinical initiators. We defined a base case concerning the mean coverage of the tumour sample (100x) and the germline reference sample (30x). We chose these coverage values as they resemble coverage values used in previous micro-costing analyses for WGS [28–30] and are similar to the average coverages for tumour sequencing (>70x-106x) reported in the established genomic programmes mentioned before [14, 18, 20, 24, 32, 33]. For each of the determined steps in the diagnostic workflow, we identified the required consumables, equipment and maintenance contracts, and personnel involved (Fig. 1). We assigned to each of these the public list prices/contractual prices without VAT and calculated the amount required per case and the resulting costs per case. Additionally, we determined which personnel are required and assigned, when possible, the mean working time (in min) per case. Utilising the personnel costs including social contributions (German tariff wages TV-L and TV-Ä, level 3; [34]) per year and working hours per year, we calculated the costs per minute for each involved position (Supplementary Table 1), for which we could determine hands-on time (technical assistants – TV-L E8/3, biologists – TV-L E13/3, physicians – TV-Ä Ä1/3 and Ä2/3). Technical assistants are responsible for the wet lab workflow (sample processing, library preparation and sequencing) in collaboration with trainee doctors responsible for tumour outlining. While bioinformaticians are developing and maintaining the computational analysis pipelines, biologists perform variant interpretation and diagnostic report generation. Medical specialists confirm the diagnostic reports and ultimately release the report. In addition, we assigned yearly costs according to the aforementioned wages to positions, for which we did not determine hands-on time (bioinformatician, case management, quality management) and divided these by the case volume (Supplementary Table 1). For computational data analysis, we identified costs for base calling licences (per base) and licences for databases used for variant interpretation. To include costs for the implementation of the WGS/WTS workflow, its further development and the continuous improvement of the pipelines for computational data analysis, we consider a flat fee for research and development of half a scientific staff position (E13/3) per year. The detailed costs per category and the positions within these categories are documented in Supplementary Table 1.

### Cost analyses and scenario design

The base case costs were calculated as follows: directly calculated per case (consumables for WGS and WTS; computational data analysis); costs per year were divided by the assumed number of diagnosed cases per year (costs for equipment maintenance; research and development). In contrast, costs for equipment were divided by its operating time and the assumed number of diagnosed cases per year. The operating time for the equipment was set to seven years, except for the sequencing platform and the DRAGEN server, which we expect to be replaced after five years. For the realistic scenario, the personnel costs were calculated by dividing wage costs per year for each required position by the case volume per year in contrast to the idealised scenario, in which we assigned personnel costs according to the hands-on time, when possible. The number of personnel considered is based on the hands-on time per case and the respective case volume per year. As redundancy for vacation or sick leave, we added 0.5x to 1x positions depending on the roles. The costs for different sequencing flow cells were calculated by dividing the maximum number of expected reads passing the filter per flow cell by the number of reads required to reach the chosen mean coverage. This value shows the maximum number of samples per flow cell. Subsequently, the list prices of the flow cell were divided by this number to identify the flow cell costs per sample. The formulas used for modelling the costs in the inflation/deflation scenarios and in the alternative coverage scenario are listed in Supplementary Table 2.

### Cost calculator

A R Shiny app [35] was developed to enable adaptation of WGS/WTS costs to different workflows based on the wet and dry lab variables identified in this article. It facilitates modelling of sequencing costs per patient depending on equipment, consumables and personnel costs, sequencing platform, flow cell type, desired mean coverage, and sample volume per year. For the WGS analysis, the app takes into account the following equation for the variables involved:

$${d}_{T}+{d}_{N}=\frac{1}{2}\times e\times {n}_{{reads}}\times {n}_{{cycles}}/\left({n}_{{patients}}\times {n}_{{genome}}\right)$$

Here, *d*_*T*_ and *d*_*N*_ are the mean coverage of the tumour and the normal samples, the factor ½ accounts for paired-read sequencing, *e* denotes the sequencing efficiency linking actual and theoretical mean coverage, *n*_*reads*_ is the total number of reads generated by the flow cell, *n*_*cycles*_ the number of sequencing cycles, *n*_*patients*_ the number of patients analysed per flow cell, and *n*_*genome*_ = 3.3 × 10^9^ the size of the human genome. In the formula above, $\frac{1}{2}\times {n}_{{reads}}\times {n}_{{cycles}}/{n}_{{patients}}$ are the number of bases that are sequenced per patient. Sequencing efficiency *e* = 1 corresponds to an ideal scenario in which all sequenced bases contribute to the coverage of the human genome. In practice, several factors, including non-human sequences and sequences shorter than the maximum read length, contribute to lowering the sequencing efficiency. The sequencing efficiency can differ for different sample types and different wet lab workflows and needs to be determined experimentally. In the current study, and as the default value for the cost calculator, the sequencing efficiency was set to *e* = 0.44, the value we observed for FFPE samples processed with the workflow of the NGS laboratory in Heidelberg.

## Results

### Cost analysis of WGS/WTS of a predefined workflow

**Fig. 2:**
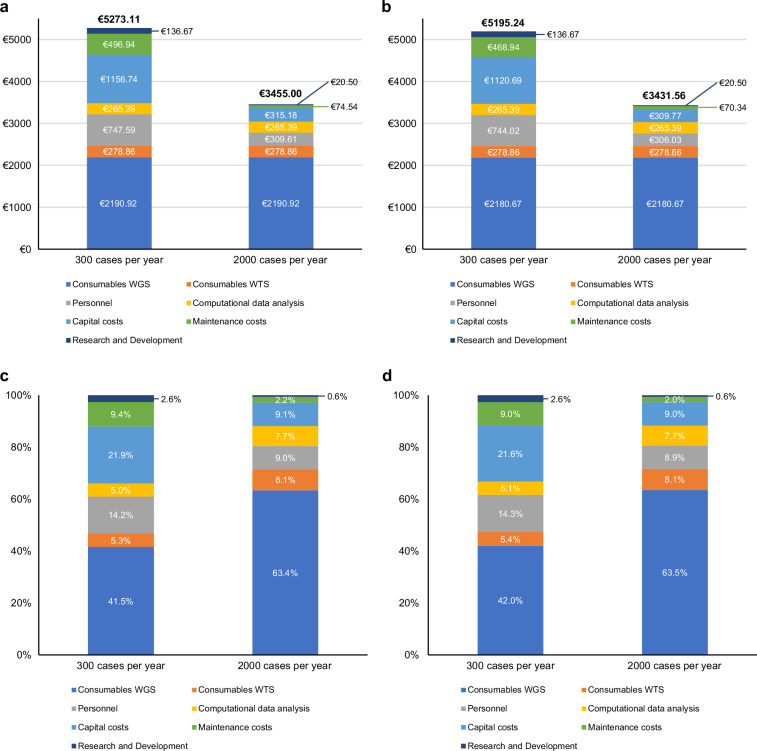
Total cost of WGS/WTS using two different wet lab protocols. Total costs of WGS/WTS (300 or 2000 diagnosed cases per year) are divided into seven categories (consumables WGS and WTS; personnel; computational data analysis; capital and maintenance costs for equipment; research and development), comparing two wet lab protocols (including or excluding DNA shearing), and their percentage contribution to the overall costs per case. **a** Total costs per case of WGS/WTS utilising a wet lab protocol, including DNA shearing and **c** the percentage contribution of each cost category to the total costs. **b** Total costs per case of WGS/WTS utilising a wet lab protocol, excluding DNA shearing and **d** the percentage contribution of each cost category to the total costs.

In order to calculate the total costs for a typical diagnostic workflow (Fig. 1), we subdivided costs into the following categories: consumables WGS, consumables WTS, equipment costs and maintenance costs, personnel, research and development, and computational data analysis. We determined the costs, excluding VAT and other aspects summarised in Table 1, in these categories for two different library preparation protocols involving either the Twist Bioscience library preparation kit (Twist Bioscience, San Francisco, CA, USA), which includes a mechanical fragmentation step, or the Illumina tagmentation kit (Fig. 2). We analysed the total costs for a volume of 300 cases per year in comparison to 2000 cases per year. We defined 300 cases because it represents the number of cases that a university medical centre will be reimbursed roughly per year in the genomDE model project in Germany [16]. The costs between the two library prep protocols only differ marginally (Fig. 2a, b), despite the requirement of additional equipment in case of the Twist library preparation protocol. The total costs per case without VAT for WGS/WTS utilising Twist amount to €5273.11 (300 cases) and €3455.00 (2000 cases) (Fig. 2a). WGS/WTS involving the tagmentation library preparation amounts to €5195.24 (300 cases) and €3431.56 (2000 cases). The amount of hands-on time in the wet lab differs only marginally between the two protocols (136.6 min vs. 130 min; see Supplementary Table 1), resulting in nearly identical personnel costs. We did not discriminate specific error rates and QC failure rates between the protocols as these mainly depend on common preanalytical parameters, e.g., sample quality and tumour cell content. Analysis of the contribution of each cost category reveals that consumables account for either ~47% or ~71% of total costs, irrespective of the chosen library preparation protocol (Fig. 2c, d). Consumables and computational data analysis are the only cost categories that do not decrease with an increase in cases per year. For the other cost categories, however, we observe significant cost reductions due to scaling effects. In the case of personnel costs, we only partially observe scaling effects as we discriminate between positions with hands-on time per case (no scaling effect) and positions for which we consider annual wages. We did not include overhead costs (e.g., electricity costs, rent; Table 1) in our main analysis, as these can substantially differ between centres. Other studies have set overhead costs to 20% additional to their total costs [29, 36]. We calculated the impact of an overhead of 20% to the costs shown in Fig. 2a (Fig. S2a, b). This increases costs either by ~ €1055 or €691, depending on the case volume per year. In conclusion, we find that consumables have the highest impact on the total costs of WGS/WTS, and their contribution increases with higher case numbers per year.

**Table 1:** Cost parameters not considered in detail in the micro-costing analysis

Cost categories	Examples
Taxes	VAT
Organisational overhead	Rent of laboratory and non-laboratory spaces, Energy costs, Water costs
Administrative overhead	Office supplies, Billing
Clinical processing	Costs for molecular tumour boards, Costs for structured documentation, Consent collection
Personnel	Education, Seminars

### Costs for sequencing flow cells have the highest impact on total costs

**Fig. 3:**
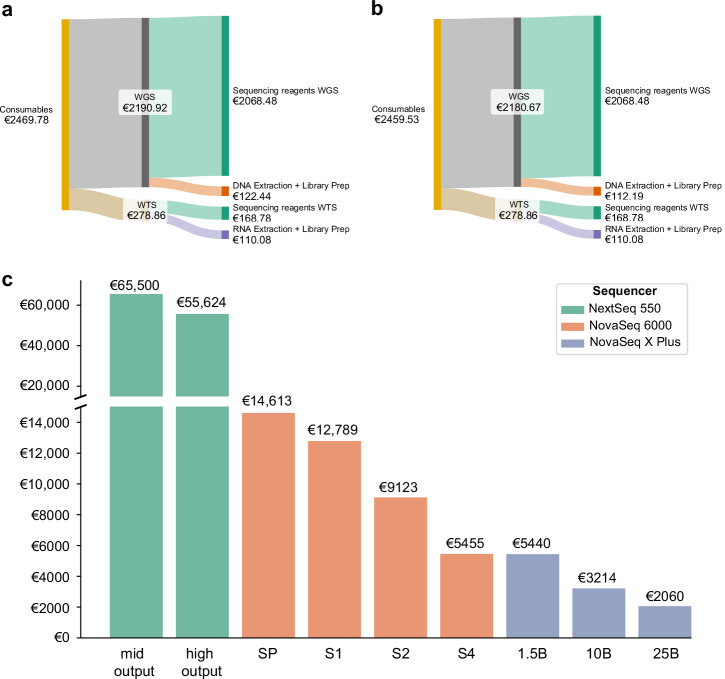
Consumable costs for WGS/WTS. Distribution of consumable costs of WGS and WTS and comparison of Illumina flow cell costs depending on the utilised sequencing system. **a** Sankey plot for consumable costs for DNA/RNA extraction, library preparation for WGS and WTS for a wet lab workflow, including DNA shearing. **b** Sankey plot for consumable costs for DNA/RNA extraction, library preparation for WGS and WTS for a wet lab workflow excluding DNA shearing. **c** Costs for flow cells utilised in WGS per case compared between Illumina flow cell sizes and Illumina sequencing systems.

As consumable costs represent the highest contributor to total costs, we analysed their composition in more detail. We subdivided the costs into consumables required for WGS or WTS. The costs for WTS consumables amount to €278.86, representing only 11.3% of total consumable costs (Fig. 3a, b). Thus, the major part of consumable costs (88.7%) stems from WGS consumables. We further subdivided the consumable costs into costs for library preparation consumables and sequencing consumables (for the Illumina NovaSeq X Plus platform). Sequencing consumables for WGS amount to €2068.48 and consumables for library preparation to €122.44 or €112.19 (Fig. 3a, b). Therefore, sequencing consumables, including the flow cell (€2060.00), represent 84% of all consumable costs. As flow cell costs account for a significant proportion of the overall costs for WGS/WTS, we analysed the costs of different flow cells, which are used on benchtop (NextSeq 550) and scalable Illumina sequencing platforms (NovaSeq 6000, NovaSeq X Plus). We determined the price per case for WGS, considering the base case values for mean coverage. As shown in Fig. 2, the flow cell used in our base case (NovaSeq X Series 25B) allows the largest number of single reads (52 billion reads) sequenced and is also the most cost-efficient flow cell in this comparison (Fig. 3c). Notably, the costs per case range from €65,500 for the mid output flow cell used in a benchtop sequencing platform to €5455 for the S4 flow cell, which is the “largest” flow cell available for the NovaSeq 6000 platform, which is still widely used in sequencing laboratories. In conclusion, the choice of the sequencing platform and the flow cell used for WGS has a significant impact on the total costs of the entire diagnostic workflow.

### A more realistic cost estimate of the WGS/WTS diagnostic workflow

**Fig. 4:**
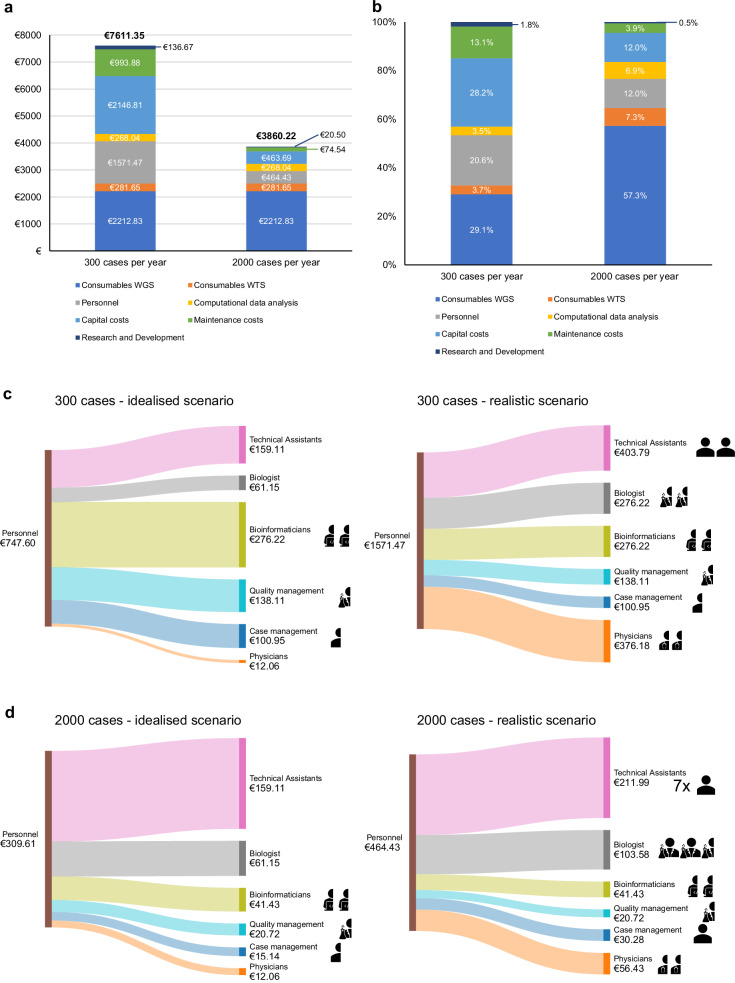
Total cost of WGS/WTS in a realistic diagnostic scenario. Total costs, percentage contribution of the cost categories and comparison of personnel cost between the idealised scenario and realistic scenario. **a** Total costs per case of WGS/WTS (300 or 2000 diagnosed cases per year) if back-up equipment and vacation/illness cover of personnel are considered. **b** Percentage contribution of cost categories to total costs from **a**). **c** Comparison (Sankey plots) of personnel costs per case (300 diagnosed cases per year) of the idealised scenario (see Fig. 2) with the realistic scenario. In the case of the idealised scenario, personnel costs of the technical assistants, biologists and physicians are calculated based on exact working time per case. Staff positions (50% or 100%) are presented by pictograms allocated to the different position types. **d** Similar to **c** comparison (Sankey plots) of personnel costs per case for a total amount of 2000 cases diagnosed per year.

The cost analysis presented so far represents an idealised scenario. It does not consider the requirement of equipment for backup and cover for personnel on sick leave or vacation. To have a more realistic view on the costs for our diagnostic workflow (focusing on the Twist protocol only), we included the costs for backup equipment and maintenance and added 1% costs for consumables and computational data analysis for an error rate of cases, which have to be re-sequenced (1 in 100 cases), which is based on the experience of our diagnostic workflow (Fig. 4). For comparison, we also calculated the impact of higher error rates (5% and 15%) on total costs, as error rates can vary between centres and sequencing protocols (Fig. S3). Additionally, we calculated costs for personnel in this scenario only per position in contrast to the prior analysis, in which we considered hands-on time per case for most of the involved roles (Fig. 4c, d). Taken these parameters together, we find the costs for 300 cases per year to be €7611.35 per case and for 2000 cases per year to be €3860.22 per case (Fig. 4a). The difference to the prior idealised scenario (Fig. 2) is €2338.24 per case (increase by 44.3%) for 300 cases per year and €405.22 per case (increase by 11.7%) for 2000 cases per year. We also calculated the total costs when considering 20% additional overhead, resulting in €9133.62/case (300 cases scenario) and €4632.26/case (2000 cases scenario) (Fig. S2c, d). In both the idealised and realistic scenarios, the costs for personnel with bioinformatic and QM expertise are the same regardless of the number of cases per year. These costs comprise half a position for pipeline development and maintenance, half a position for database development and maintenance, and a half-time position for quality management, including management of accreditation and audits and participation in round-robin tests (Fig. 4c, d). In addition, we included a 0.5–1 position for case management (same tariff level as technical assistants), depending on the scenario and the case volume. In the realistic scenario, we added one half-time or full-time position for technical assistants and biologists, depending on the case volume per year, as cover for sick leave or vacation. This increases the costs for technical assistance from €159.11 per case to €403.79 (300 cases) and €211.99 (2000 cases), and the costs for biologists from €61.15 per case to €276.22 (300 cases) and €103.58 (2000 cases) (Fig. 4c, d). In total, costs for personnel increase from €747.59 to €1571.47 (300 cases) and from €309.61 to €464.43 (2000). In summary, assuming the costs for WGS/WTS to be more realistic results in a substantial increase (low case volume) and a moderate increase (high case volume).

### Impact of inflation/deflation and an increase in mean coverage

**Fig. 5:**
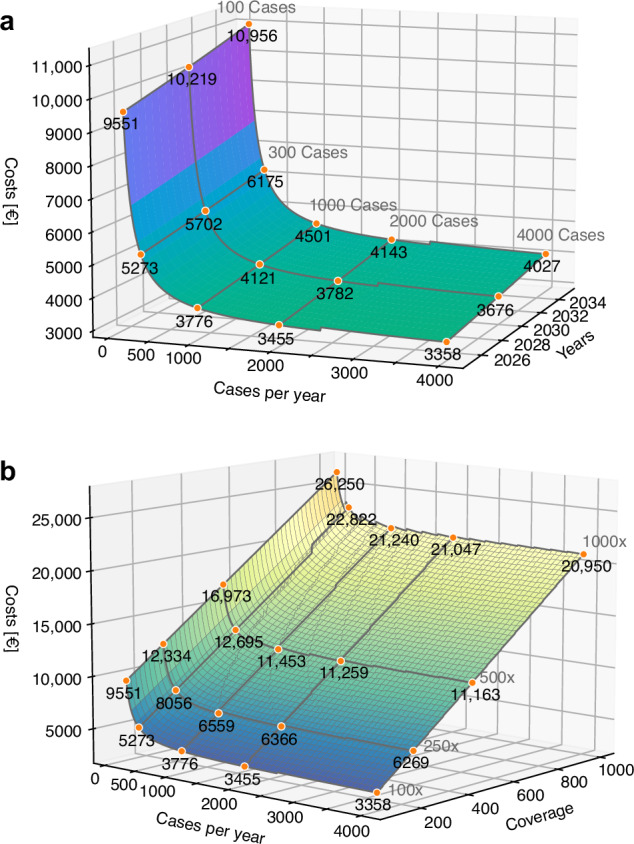
Inflation and coverage scenario. Total costs per case in relation to case numbers diagnosed per year (100–4000 cases per year) and an inflation rate of 2% or in relation to the mean coverage. **a** Total costs modelled with an inflation rate of 2% per year over a timespan of ten years (2025–2035). **b** Total costs in relation to case numbers per year and an increase in mean coverage (100x to 1000x).

An important economic aspect in the analysis of WGS/WTS costs is the impact of increasing or decreasing costs over time, i.e., due to inflation and deflation. Therefore, we modelled the influence of 2% inflation for a decade in comparison with the case volume (range 100–4000 cases) (Fig. 5a). Moreover, we modelled other rates of inflation and deflation (2%, 5%, 10%) for a period of 5 years (Fig. S4). In case of inflation, we did not apply the rates to the equipment costs, as equipment is bought once prior to the observed time frame. For simplicity, we also did not include the costs for the replacement of the equipment after the end of the operating time. Modelling of the case volume shows a sudden increase in costs at 2500 cases per year, which eventually flattens out. This is due to the maximum capacity of the sequencing platform, which is at 2500 cases per year, and thus a second platform has to be considered at higher number of cases. The model reveals that a rate of 2% inflation increases the total costs by ~7–9.5% after five years and ~14.7–19.9% after 10 years (Fig. 5a). When modelling the deflation scenarios, we did not apply the rates to the equipment costs as well as to personnel costs, as tariff wages are usually not decreasing during deflation. Cost reduction ranges from a few hundred to more than €1000.00 per case, depending on the applied rate and the case volume (Fig. S4b). We modelled a different scenario in which the mean coverage of tumour samples (WGS) increased (Fig. 5b). A higher mean coverage allows a more sensitive and accurate variant calling, especially in cases with low tumour purity. Again, we compared the increase (range 100x to 1000x) with a change in case volume. The increase in mean coverage mainly influences the flow cell costs, as fewer cases can be sequenced using one flow cell and the costs of computational data analysis and storage. In addition, a higher coverage lowers the maximum capacity of the sequencing platform, resulting in an earlier requirement for additional sequencing platforms. For example, with a mean coverage of 500x, the maximum capacity is 500 cases per year. Therefore, eight sequencing platforms are required to diagnose 4000 cases per year at this coverage. In general, it can be stated that increasing the mean coverage substantially influences the total costs as a mean coverage of 500x more than doubles the costs within the range of 300–4000 cases. The highest mean coverage, which we used in our model (1000x), results in total costs of more than €20,000 irrespective of the number of cases diagnosed per year. This substantial impact is due to the major contribution of flow cell costs to the total costs shown in Figs. 2 and 3. In summary, our scenario analyses show that a higher mean coverage in WGS influences total costs significantly, as it directly affects the sequencing consumable costs.

### Cost calculator

The WGS/WTS costing model can be individualised using the publicly available cost calculator that was developed in parallel (https://hd-molpath.de/WGS-cost-calculator). The web app, which will be maintained and updated on a regular basis, considers various key contributing variables and computes the costs per patient as output. These variables include equipment, consumable and personnel costs, the number of patients sequenced per year, the mean coverage for tumour and normal samples for WGS and the total number of reads for WTS. To model the coverage, a theoretical value is calculated from flow cell characteristics that is subsequently corrected to the actual value by a lab-specific efficiency factor, as detailed in the methods section. The latter is influenced by the level of DNA fragmentation, the proportion of reads not mapping to the human genome and the proportion of reads removed by deduplication. For the previously discussed scenario of a FFPE tumour sample analysed with a 25B flow cell and 300 sequencing cycles in our own lab, the efficiency factor was 0.44.

## Discussion

In this study, we provide a comprehensive in-depth costing model for performing WGS and WTS using short-read technology in routine clinical care. Previously, WGS using FFPE material was considered to be not valuable in the clinical setting [37]. However, recent reports highlight the general feasibility and demonstrate the applicability of FFPE-based WGS in actionable variant detection [38, 39]. Thus, we focused on short-read sequencing due to its wide availability and broad applicability for both fresh and FFPE input material. Similar to previous micro-costing studies focusing on gene panels to WGS [28–30, 36, 40], we categorised costs into those for consumables, personnel, equipment and computational data analysis. When comparing the base case scenario with previous analyses, our total costs are within the range of the reported costs [28, 29, 40] or even closely match these [30], despite differences in cost structures, e.g., national differences in wages or centralised sequencing versus federalised sequencing in centres across the country.

We found that consumables, especially sequencing consumables, are the main driver of total costs. This is concordant with a recently reported micro-costing analysis of targeted gene panels in Norway [36] and previous analyses focusing on WGS [28, 30, 40]. As our base case was based on using the Illumina flow cell with the highest throughput, we did not observe scaling effects when increasing the case volume. As we found the flow cell cost per case to be €2060 for WGS and total costs per case ranging from €7611 (realistic scenario, 300 cases) to €3455 (idealised scenario, 2000 cases), we are still far away from achieving the widely acclaimed goal of the ‘$1000’ genome in the field of clinical cancer diagnostics [31]. In consequence, decreasing consumable costs by innovation of new sequencing technologies or more competitors entering the sequencing market are paramount to facilitate the broad implementation of WGS/WTS in routine cancer care.

We are aware that our cost modelling reflects specific assumptions regarding positions and roles of personnel, wage rates specific to Germany, and, e.g., assumptions concerning the lifespan of devices, and workflow design, which may be different in other lab scenarios and countries. However, the comparison of our results to the aforementioned previously published micro-costing analyses shows that albeit these analyses are based on different assumptions and prerequisites, their findings are similar. In the analyses by Thangavelu et al. [30] and Gordon et al. [41], consumable costs dominate total costs even higher, ranging from 76% (2500 case volume per year) [30] to 86% (100 cases and personnel costs included) of total costs [41]. In our base case, personnel costs contribute from 9.0% to 14.2% to total costs depending on the sample volume, which is in a similar range (2.8% to 15%) as reported in the previously published analyses, despite different workflows and roles of the personnel [28–30]. Thus, our WGS/WTS cost analysis comes to similar conclusions, demonstrating its general applicability. Importantly, our cost calculator further enhances the transferability as the tool allows a multiparametric adjustment to specific requirements of other countries, diagnostic centres and contexts.

As previously reported, we observed significant reductions in total costs due to scaling effects in other cost categories, such as personnel and equipment [29, 36, 40]. However, these scaling effects reach a plateau when the case volume exceeds 2000 cases, as further increases in case throughput only marginally reduce costs. This highlights that the implementation of WGS/WTS in clinical routine should be accompanied by a high demand and, consequently high case volume per year. Additionally, high throughput increases the likelihood of substantial discounts being negotiated with manufacturers and other suppliers. We excluded vendor discounts as these heavily depend on individual negotiations between the diagnostic centres and the involved companies and are thus difficult to predict and to generalise. However, our cost calculator enables the inclusion of discounts for individual centres by changing the relevant cost parameters. In contrast, small diagnostic centres processing case numbers ranging from 100 to 300 per year on smaller sequencing platforms face comparably high costs per case, potentially exceeding €10,000. In diagnostic facilities, a variety of diagnostic options, including small and large gene panels and WES, could be offered, which could potentially cross-finance the provision of WGS.

In our base case, we considered a mean coverage of 100x for WGS on tumour material and 30x for the reference sample, which are comparable to the base case assumptions in other micro-costing analyses of WGS [28, 30]. As these values represent the minimum requirement for faithful variant detection, a higher mean coverage could improve the diagnostic value of WGS/WTS, especially when considering samples with low tumour cell content (e.g., PDAC). We found that considering a mean coverage of 250x, which allows accurate variant identification when confronted with low tumour purity, already substantially increases total costs (1.29x to 1.86x of the base case depending on case volume), mainly driven by flow cell costs. This is another compelling reason for a decrease in consumable costs, democratising the application of WGS/WTS in molecular cancer diagnostics.

Furthermore, we modelled the impact of inflation on total costs. We chose a range between 2% and 10% inflation as inflation in Western countries varied within this range in the last decades and the rate of 2% inflation represents the target rate of the European Central Bank [42]. It is important to consider and prepare for potential future cost increases, for example, due to rising energy costs. For comparison, we also modelled deflation using the same range of rates. Deflation especially plays a role in light of consumable costs, which have steadily decreased over the last few decades. Of note, the emergence of new sequencing methods, if applicable, or other innovations such as new sequencing platforms that substantially increase throughput could potentially decrease sequencing costs at a higher rate or even exponentially. A recent analysis by Ehman et al. shows that the sequencing costs per genome (Illumina short-read sequencing) have dropped substantially with the market release of new sequencing platforms (HiSeq, NovaSeq 6000 and NovaSeq X Plus) between 20.8% and 45.8% [43]. In our analysis, we see a drop of 62.2% costs per flow cell when comparing the largest flow cell for the Illumina NovaSeq 6000, which was introduced in 2017, with its successor, the Illumina NovaSeq X platforms, which were introduced in 2023. As, on average, Illumina releases a new sequencing machine every 6.5 years, we could assume the costs for the flow cell and therefore sequencing costs to drop by half in 2029. However, the technological advancement and innovation in sequencing technology are difficult to predict, as demonstrated by the cost analysis published by the National Human Genome Research Institute [44]. In this analysis, the costs for sequencing a human genome were analysed between 2001 and 2022 and outperformed Moore’s Law starting from 2008 when NGS replaced Sanger sequencing at the sequencing centres, underscoring the difficulty to predict cost developments in the future.

For comparison to our idealised base case, we analysed a more realistic scenario in which we considered sick leave and holiday cover for personnel, an error rate in sequencing, and backup equipment. These conditions result in a substantial increase in total costs per case, particularly in the scenario of 300 cases per year. We are aware that allocating all personnel costs to a single diagnostic workflow does not accurately reflect reality, given that staff perform additional tasks and are involved in various diagnostic workflows besides WGS/WTS. Nevertheless, we believe that our calculations provide a meaningful approximation of the actual costs.

Automating the labour-intensive library preparation for sequencing could potentially decrease personnel costs for technical assistants [45]. We and others have previously reported successful automation of DNA and RNA library preparation using liquid handling robots [46, 47], highlighting the feasibility and potential of integrating automation into WGS/WTS workflows. Besides wet lab automation, variant interpretation of the processed sequencing data, performed by trained biologists in our workflow, could be accelerated by incorporating artificial intelligence (AI) assistance [48], resulting in a further decrease in personnel costs and shorter turnaround time. However, a broad integration and scalability of AI in computational analysis of WGS/WTS data requires the shift from local data analysis and storage to cloud-based solutions. These cloud-based solutions could offer further cost-reduction potential in the future, especially with increasing case numbers and consequently a steady increase in data size. Further areas for cost reductions are the choice of variant database used for variant interpretation and the choice of library preparation protocols requiring less equipment. We modelled the latter by our comparison between the Twist library preparation and the tagmentation library preparation, which does not require mechanical shearing. However, in both cases, only minor cost savings are achieved as these costs contribute between 0.6% to 2.3% to the total costs.

CGP from large gene panels to WGS/WTS is usually followed by an interdisciplinary discussion of the results within a molecular tumour board (MTB) and recommendations of matched targeted therapies [49]. Of note, we did not include costs associated with MTBs and other aspects summarised in Table 1 in our analysis as we solely focused on the diagnostic workflow from sample processing to diagnostic report generation. Although these factors affect budget calculations and reimbursement rate negotiations, they encompass costs that either fluctuate significantly between sites (e.g., rent and energy expenses) or are challenging to predict (e.g., education and personnel development expenses). Some previous micro-costing studies have summarised these costs as overheads, adding a percentage (20%) to the total [29, 36]. For comparison, we modelled the impact on total costs of a similar overhead, thereby addressing aspects to which we did not directly assign costs.

In conclusion, we have dissected and modelled the costs of a clinical-grade WGS/WTS diagnostic workflow, identifying key contributors. This serves as a landmark costing guide for diagnostic centres currently implementing, or planning to implement WGS/WTS in their clinical routine, and illustrates opportunities for cost reductions. Sequencing consumables are the most interesting and promising target for cost reductions on a global level. Integrating automation is another promising area for future efficiency gains and, consequently, reduced costs in the long term. Ultimately, sinking costs will lead to a broad implementation of WGS/WTS while simultaneously lowering the costs through increased throughput. This self-reinforcing cycle could elevate WGS/WTS to the standard procedure in molecular cancer diagnostics, thereby widening access for patients benefiting from CGP.

## Supplementary information

Supplementary Information

Data Set 1

Data Set 2

## Data Availability

Data used in this study are available in the supplementary information of this article.
