# Supplementary material for: Implementing whole genome and transcriptome sequencing for cancer patients in routine healthcare: a comprehensive guide to costing
Source: Br J Cancer. 2026 Apr 7;134(12):1810–9. doi: 10.1038/s41416-026-03422-0 (PMC13226723; doi:10.1038/s41416-026-03422-0)
Supplement: Supplementary file 1 — Supplementary Information [file 41416_2026_3422_MOESM1_ESM.pdf]

Supplementary Information

Supplementary Figures 1-4 (pages 1-4)

Supplementary Tables 1-2 (Descriptions page 5)

Supplementary Figures

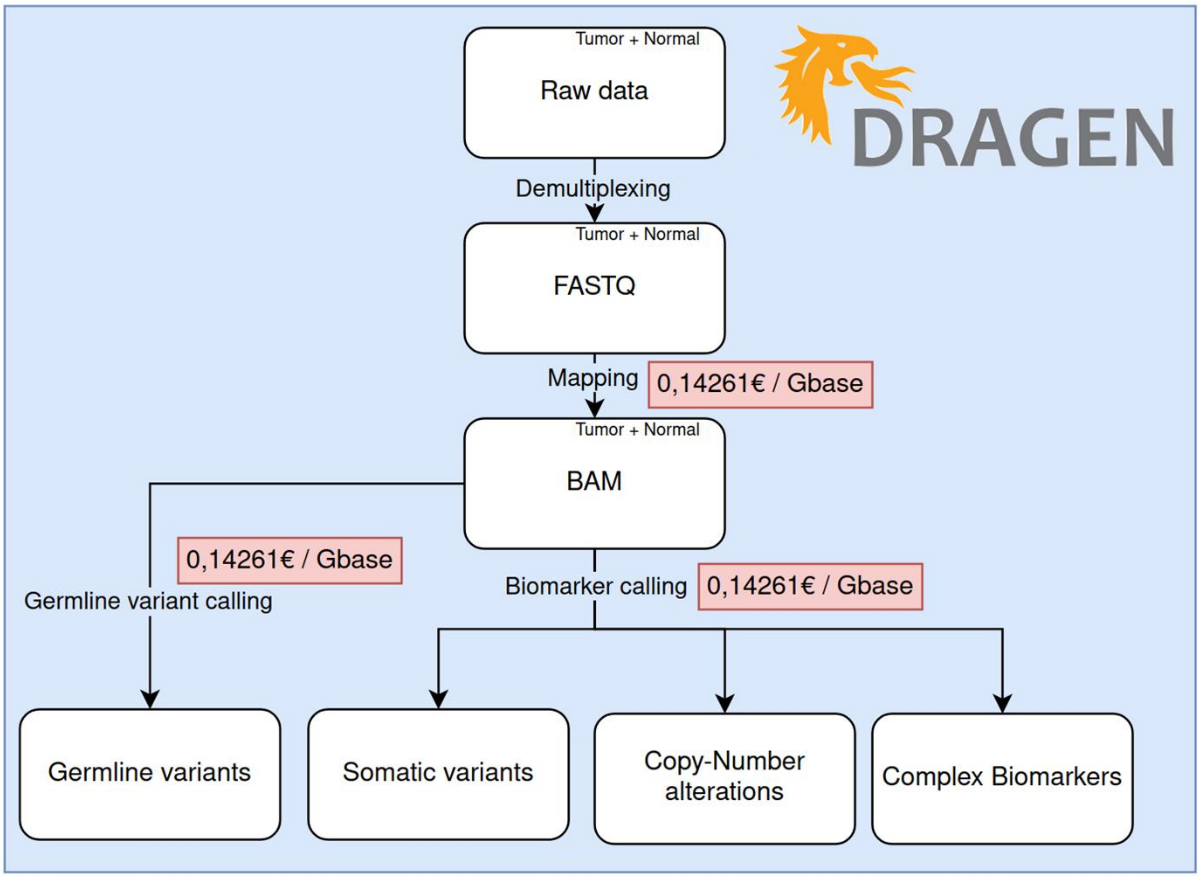

Supplementary Figure 1. Computational analysis pipeline.

Schema describing the computational analysis pipeline utilizing a local DRAGEN setup. The pipelines include demultiplexing of the raw data, mapping, and calling of variants, copy-number alterations and complex biomarkers. Mapping, germline variant calling and biomarker calling are steps with assigned license costs per Gbase.

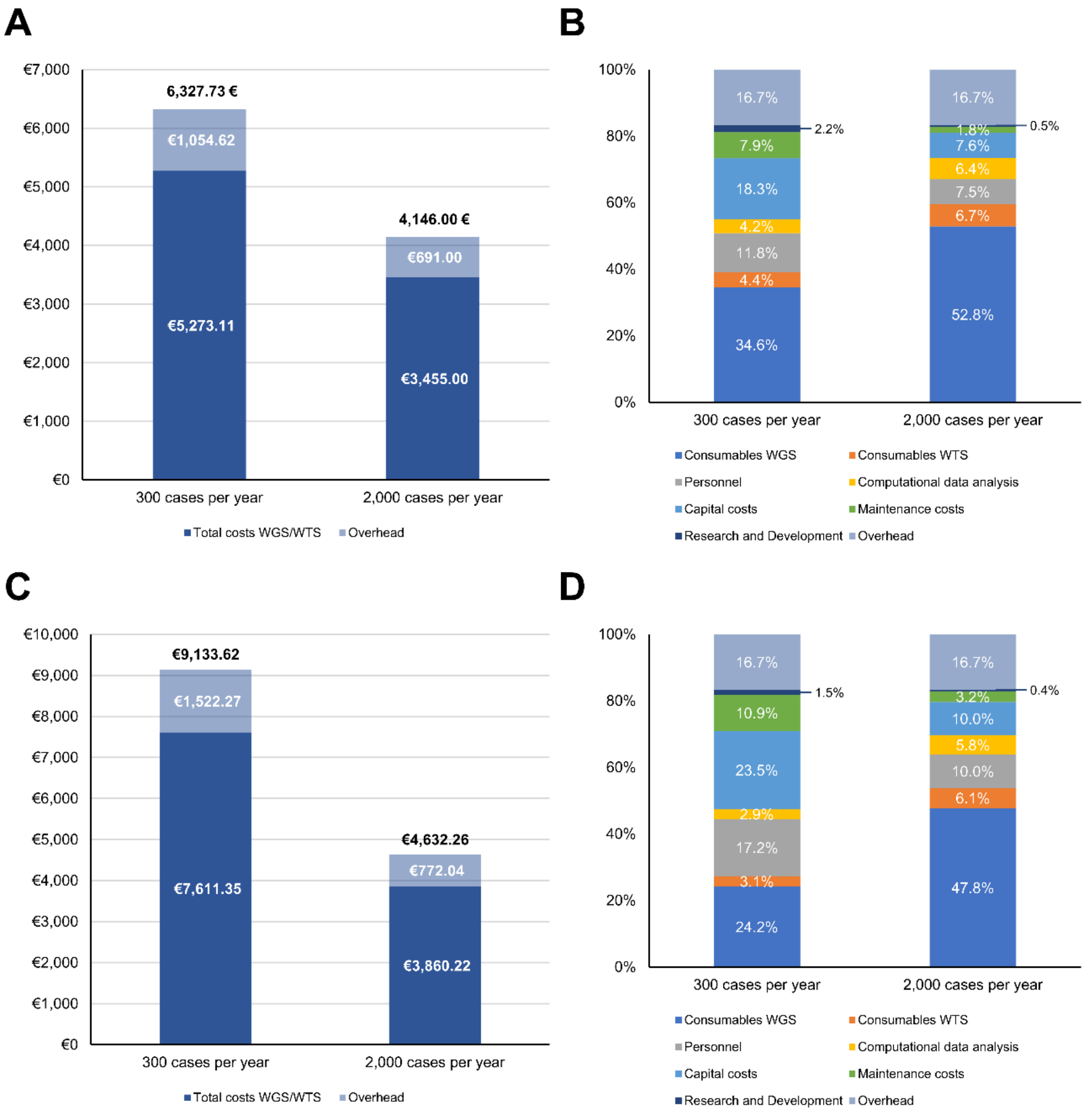

Supplementary Figure 2. Total costs including an overhead of 20%.

Total costs, percentage contribution of the cost categories when 20% overhead costs (see table 1) are considered. **A)** Total costs including overhead of WGS/WTS in the idealised scenario (for comparison see Figure 2A). **B)** Percentage contribution of all cost categories and overhead to the total costs in A). **C)** Total costs including overhead of WGS/WTS in the realistic scenario (for comparison see Figure 4A). **D)** Percentage contribution of all cost categories and overhead to the total costs in C).

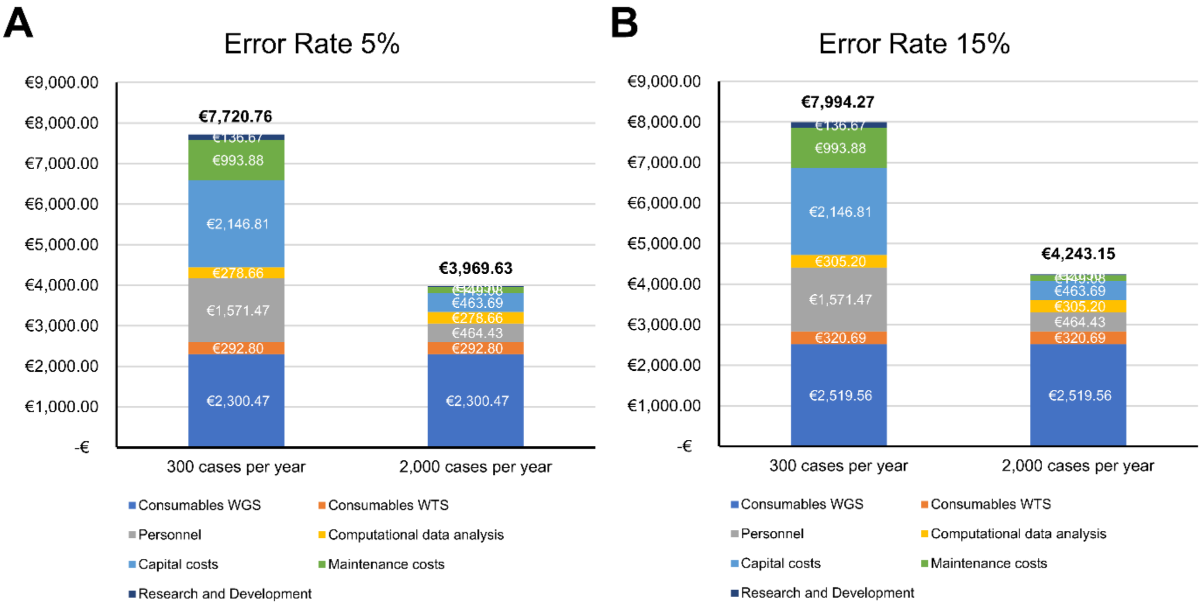

Supplementary Figure 3. Impact of 5% and 15% error rates on total costs in the realistic scenario.

Total costs of WGS/WTS, when considering an error rate of 5% and 15%. **A)** Total costs in case of 5% error rate. **B)** Total costs in case of 15% error rate.

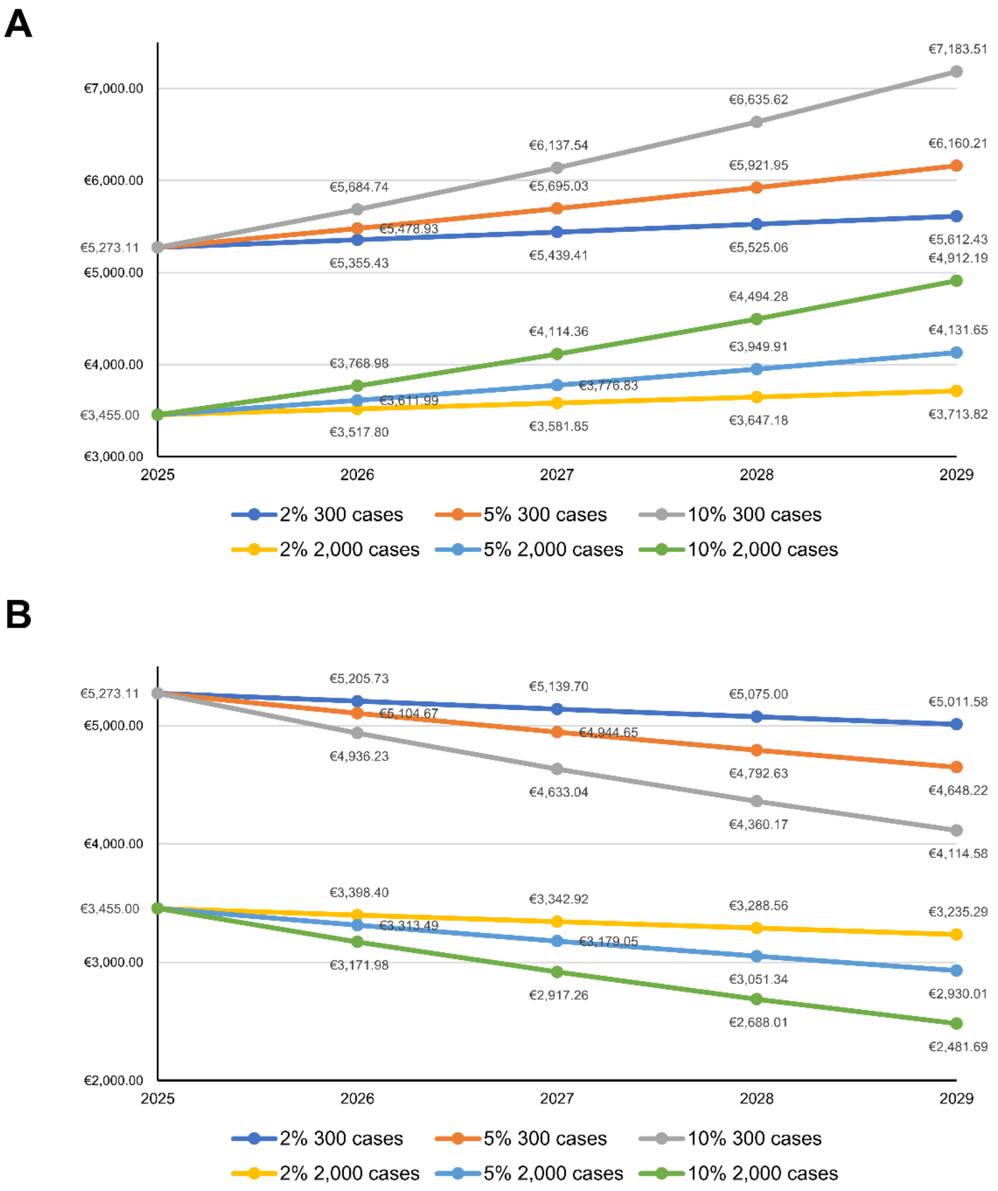

Supplementary Figure 4. Inflation and Deflation scenarios.

Impact of inflation or deflation on the total costs from 2025 to 2029. **A)** Impact of inflation rates of 2%, 5% and 10% on total costs of WGS/WTS except capital costs for equipment, which are excluded from the increase of costs. **B)** Impact of deflation rates of 2%, 5% and 10% on total costs of WGS/WTS except capital costs for equipment and personnel costs, which are excluded from the decrease of costs.

Supplementary Tables

Supplementary Table 1 – Raw data

All wet and dry lab variables and their default values used for calculating WGS/WTS total costs in this article.

Supplementary Table 2 – Formulas

Formulas used for the inflation and coverage scenarios shown in main figure 5.
